# Aberrant expression of the *PHF14* gene in biliary tract cancer cells

**DOI:** 10.3892/ol.2013.1278

**Published:** 2013-03-29

**Authors:** TAKAKO AKAZAWA, KOHICHIROH YASUI, YASUYUKI GEN, NOBUHISA YAMADA, AKIRA TOMIE, OSAMU DOHI, HIRONORI MITSUYOSHI, NOBUAKI YAGI, YOSHITO ITOH, YUJI NAITO, TOSHIKAZU YOSHIKAWA

**Affiliations:** Department of Molecular Gastroenterology and Hepatology, Graduate School of Medical Science, Kyoto Prefectural University of Medicine, Kyoto 602-8566, Japan

**Keywords:** *PHF14*, biliary tract cancer, deletion

## Abstract

DNA copy number aberrations in human biliary tract cancer (BTC) cell lines were investigated using a high-density oligonucleotide microarray. A novel homozygous deletion was detected at chromosomal region 7p21.3 in the OZ cell line. Further validation experiments using genomic PCR revealed a homozygous deletion of a single gene, plant homeodomain (PHD) finger protein 14 (*PHF14)*. No *PHF14* mRNA or protein expression was detected, thus demonstrating the absence of *PHF14* expression in the OZ cell line. Although the PHD finger protein is considered to be involved in chromatin-mediated transcriptional regulation, little is known about the function of *PHF14* in cancer. The present study observed that the knock down of *PHF14* using small interfering RNA (siRNA) enhanced the growth of the BTC cells. These observations suggest that aberrant *PHF14* expression may have a role in the tumorigenesis of BTC.

## Introduction

Biliary tract cancers (BTCs) are a heterogeneous group of tumors arising from the epithelial cells of the intra- and extra-hepatic bile ducts and gallbladder (1,2). Histologically, the majority of BTCs are adenocarcinomas and have a poor prognosis. The majority of BTC patients exhibit an unresectable disease at the time of diagnosis due to the advanced cancer stage. Although patients rarely have identical risk factors, it is clear that the disorders that cause chronic inflammation of the biliary tract, including primary sclerosing cholangitis, gallstones and bile duct stones, are associated with an increased incidence of BTC.

Little is known about the molecular pathogenesis of BTC (1,2). Although alterations in a number of cancer-associated genes, including *p53* and *KRAS*, have been identified as potential risk factors, the frequency of these alterations is low. Interleukin 6 (IL-6), an inflammatory cytokine, appears to have a more definite role in the pathogenesis of BTC. The activation of EGFR, ERBB2 and HGF has also been reported in BTC (1,2).

Homozygous deletions have been useful in the positional cloning of a number of tumor suppressor genes. Using high resolution single nucleotide polymorphism (SNP) arrays, we previously detected novel regions of homozygous deletions and identified potential tumor suppressor genes in human cancers (3,4). In the present study, DNA copy number aberrations in human BTC cell lines were investigated using SNP arrays to identify the genes potentially involved in BTC. It was observed that a novel homozygous deletion at the chromosomal region 7p21.3 occurred in a BTC cell line and that the plant homeodomain (PHD) finger protein 14 (*PHF14)* gene, which lies within the 19p13.2 chromosomal region, was homozygously deleted. The present study also further examined whether defective *PHF14* expression has a functional role in BTC cells.

## Materials and methods

### Cell lines

The following eight human BTC cell lines were studied: TFK1, HuCCT1, OCUG1, NOZ, OZ, SSP25, HuH28, and TKKK. These cell lines were obtained from the Health Science Research Resources Bank (Osaka, Japan) and the American Type Culture Collection (Manassas, VA, USA). The cells were maintained in Dulbecco’s modified Eagle’s medium supplemented with 10% fetal calf serum. This study was approved by the Ethics Committee of Kyoto Prefectural University of Medicine, Kyoto, Japan.

### SNP array analysis

DNA copy number changes were analyzed using the GeneChip Mapping 250K Sty array (Affymetrix, Santa Clara, CA, USA) according to the manufacturer’s instructions, as previously described (3–5). Briefly, 250 ng genomic DNA was digested with a restriction enzyme, then ligated to an adaptor and amplified by PCR. The amplified products were fragmented, biotinylated and hybridized to the microarrays. Hybridization was detected by incubation with a streptavidin-phycoerythrin conjugate and scanning of the array. Following the appropriate normalization of the mean array intensities, signal ratios were calculated between the BTC cell lines and the anonymous normal references. Copy numbers were then inferred from the observed signal ratios based on the hidden Markov model using Copy Number Analyzer for Affymetrix GeneChip mapping arrays (CNAG) software (available at http://www.genome.umin.jp).

### PCR analysis

Conventional PCR was performed using Ex Taq DNA polymerase (Takara, Otsu, Japan) according to the manufacturer’s instructions. Genomic DNA and mRNA were quantified using the real-time fluorescence detection method, as described previously (5). The primers that were used for the PCR are shown in Table I. The endogenous controls for the mRNA and genomic DNA levels were *GAPDH* and long interspersed nuclear element-1 (LINE-1), respectively.

### Immunoblotting

Immunoblots were prepared according to previously published methodology (5). Cell lysates (20 *μ*g protein per sample) were separated via SDS-polyacrylamide gel electrophoresis using 10% acrylamide gels. The anti-PHF14 rabbit polyclonal antibody and the anti-β-actin mouse monoclonal antibody were purchased from Sigma-Aldrich (Tokyo, Japan). The anti-PHF14 and anti-β-actin antibodies were used for immunoblotting at dilutions of 1:400 and 1:5,000, respectively. The anti-mouse or anti-rabbit IgG (Amersham, Tokyo, Japan) used for secondary immunodetection was diluted to 1:5,000. Antibody binding was detected using an ECL system (Amersham).

### RNA interference (RNAi)

To knock down *PHF14* expression in the cells, two small interfering RNA (siRNA) duplex oligoribonucleotides targeting *PHF14* [PHF14 Stealth Select RNAi™ siRNA HSS114491 (siRNAb) and HSS114492 (siRNAc)] and negative control siRNA duplexes were purchased from Invitrogen (Carlsbad, CA, USA). The siRNAs were delivered into OCUG1 cells using Lipofectamine RNAiMAX (Invitrogen), according to the manufacturer’s instructions. The cell viability was assessed by measuring 3-(4,5-dimethylthiazol-2-yl)-2,5-diphenyltetrazolium bromide (Nacalai Tesque, Kyoto, Japan) dye absorbance (MTT assay), according to the manufacturer’s instructions, at 24, 48 and 72 h after siRNA transfection.

### Statistical analysis

Differences between the groups were evaluated using the Student’s t-test. The statistical analyses were performed on SPSS 15.0 software (SPSS Inc., Chicago, IL, USA). P<0.05 was considered to indicate a statistically significant difference.

## Results

### Overview of genomic changes in BTC cell lines

**Figure 1:**
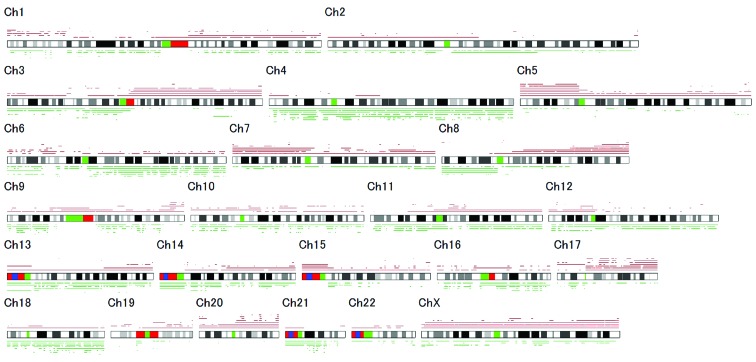
Summary of the genetic imbalances detected in eight BTC cell lines using SNP array analyses. The 22 autosomes and X chromosome are represented by ideograms showing G-banding patterns. Copy number gains are indicated by red horizontal lines above the chromosome ideogram; high-level gains (amplifications) are shown as bright red lines, whereas simple gains are shown as dark red lines. Copy number losses are indicated by green lines under the chromosome ideogram. Each horizontal line represents an aberration detected in a single BTC cell line. BTC, biliary tract cancer; SNP, single nucleotide polymorphism.

To identify the genes involved in BTC, eight BTC cell lines were screened for DNA copy number aberrations using SNP array analysis. The genetic changes that were detected are shown in Fig. 1. Chromosomal regions frequently involved in the gain of DNA were identified at 5p and 17q (seven cases, 88%), as well as 8q (six cases, 75%). The chromosomal regions most frequently associated with DNA loss were identified at 4p and 4q (seven cases, 88%) and 6q (six cases, 75%). The homozygous deletions and chromosomal amplifications are shown in Table II. SNP array analyses successfully identified chromosomal amplification regions containing known oncogenes, including *KRAS* (12p12.1) and *ERBB2* (17q12), as well as chromosomal homozygous deletion regions containing known tumor suppressor genes, including *FHIT* (3p14.2), *CDKN2A* (9p21), *CDKN2B* (9p21) and *WWOX* (16q23.1; Table II). Of these chromosomal regions, the homozygous deletion at 7p21.3 became the focus for further investigation as it was a novel alteration in BTC.

### Identification of homozygous PHF14 gene deletion

**Figure 2:**
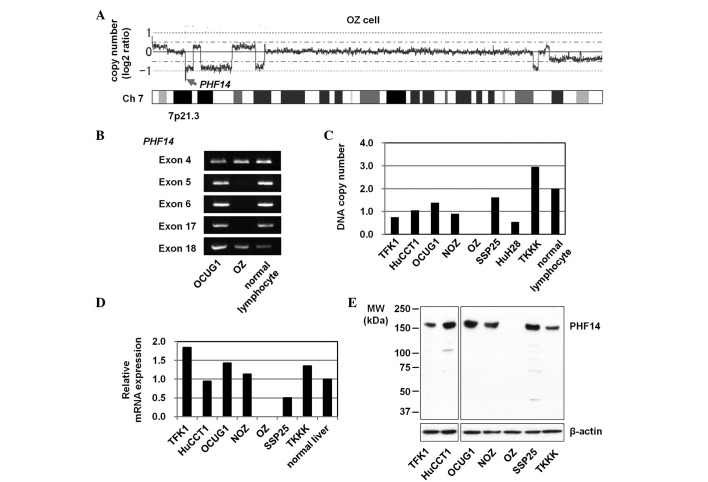
Homozygous deletion of the *PHF14* gene in the OZ cell line. (A) Chromosome 7 cytoband map and copy numbers were determined via SNP arrays of OZ cells. The arrow indicates the locus of the homozygous deletion at position 7p21.3. (B) PCR analysis of the exons in the *PHF14* gene from genomic DNA templates derived from two BTC cell lines and normal lymphocytes. (C) Copy numbers of the *PHF14* gene in the BTC cell lines, as measured by real-time quantitative PCR with reference to the LINE-1 control. Values are normalized such that the copy number in the genomic DNA derived from the normal lymphocytes was assigned a value of 2. (D) Relative expression levels of *PHF14* mRNA as evaluated by real-time quantitative RT-PCR. Results are presented as the expression level of the *PHF14* gene relative to a reference gene (*GAPDH*) in order to correct for variations in RNA amounts. Values are normalized such that the mRNA derived from normal liver samples had a value of 1. (E) Immunoblot analyses of PHF14 protein levels in the indicated cell lines, with β-actin as an internal control. *PHF14*, plant homeodomain finger protein 14; SNP, single nucleotide polymorphism.

Among the eight cell lines screened, the OZ cell line (6) exhibited a homozygous deletion at chromosomal region 7p21.3 (Fig. 2A). It was estimated that the region of deletion included five genes. Further validation experiments using genomic PCR revealed a homozygous deletion of a single gene, *PHF14*. The extent of the homozygous deletion was narrowed down to a location between exons 5 and 17 of the *PHF14* gene (Fig. 2B).

### Copy number and expression of PHF14 gene in BTC cell lines

The DNA copy numbers and expression levels of the *PHF14* gene in the BTC cell lines and control normal lymphocytes or liver (Fig. 2C–E) were then analyzed. Real-time quantitative reverse transcription (RT)-PCR and immunoblot analyses did not detect *PHF14* mRNA or protein expression, respectively (Fig. 2D and E), thus demonstrating the absence of the *PHF14* gene from the OZ cell line.

### Enhanced growth of BTC cells by PHF14-knockdown

**Figure 3:**
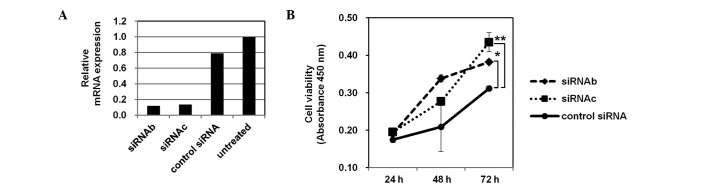
Enhanced growth of BTC cells by *PHF14*-knockdown. The BTC cell line OCUG1 was treated with two independent siRNAs targeting *PHF14* (siRNAb and sRNAc), control siRNA or left untreated. (A) Expression levels of *PHF14* mRNA were determined by real-time quantitative RT-PCR. The cells were harvested at 48 h post-transfection. (B) The effect of the siRNA targeting of *PHF14* on cell proliferation was measured with the MTT assay at the indicated times following transfection. Each assay was performed in triplicate. Values are represented as the mean ± SD. Differences were evaluated with the Student’s t-test (^*^P<0.05, ^**^P<0.01). BTC, billiary tract cancer; *PHF14*, plant homeodomain finger protein 14; siRNA, small interfering RNA.

To determine whether the defective expression of *PHF14* had a functional role in the BTC cells, *PHF14* expression was knocked down with two independent siRNA molecules (siRNAb and siRNAc) in OCUG1 cells (Fig. 3A). The *PHF14*-knockdown led to an upregulation of cell growth, as determined via the MTT assay 72 h after the transfection with siRNAb and siRNAc (Fig. 3B). These observations suggest that the defective expression of *PHF14* may promote the proliferation of BTC cells.

## Discussion

In the present study, a novel homozygous deletion at chromosomal region 7p21.3 was identified in the OZ cell line, a human BTC cell line that was established from the ascites of a patient with mucin-secreting BTC in the hepatic hilus (6). Subsequent detailed analyses revealed that the homozygous deletion was located between exons 5 and 17 of the *PHF14* gene. Moreover, the present data suggest that the defective expression of *PHF14* may promote the proliferation of the BTC cells.

Based on the amino acid sequence homology, *PHF14* is considered to be a PHD finger protein. The PHD finger protein is known to be involved in chromatin-mediated transcriptional regulation (7–9). The PHD finger domain recognizes the methylation status of histone lysine residues, including histone H3 trimethylated at lysine 4, which is associated with an ‘open’ chromatin structure and transcriptional activation. Mutations, deletions and chromosomal translocation in the genes encoding PHD finger proteins, such as the tumor suppressor ING1, have been associated with various types of cancer (8). A mutation in *PHF14* was previously identified in a colon cancer cell line (10). However, the function of PHF14 has remained unknown. Phf14, a mouse homologue of PHF14, was identified as a novel transcriptional factor that acts as a negative regulator of platelet-derived growth factor receptor-α (PDGFRα) expression in mouse mesenchymal cells (11). Furthermore, *Phf14*-null mice exhibited interstitial pulmonary hyperplasia. Mesenchymal fibroblasts derived from the *Phf14*-null mice showed an increased proliferation rate, accompanied by the enhanced expression of PDGFRα (11). The increased growth of *Phf14*^−/−^ mesenchymal cells supports the present observation that the knockdown of *PHF14* enhances the growth of BTC cells. Although the mechanisms by which *PHF14* functions in tumors remain to be elucidated, the present data suggest that alterations in the expression of *PHF14* may be involved in the tumorigenesis of BTC.

## Figures and Tables

**Table I:** Primers used for PCR.

*PHF14*	STS-marker	Forward primer	Reverse primer
Exon 4		5′-TTGGAAATGCATATAATAATGTTTAAG-3′	5′-AGCCACAGTCAGCCATTTCT-3′
Exon 5		5′-TTCTTTTTCTTTTGTGATTTTATGTGA-3′	5′-AGGGAAGTCAAAGGCAGACA-3′
Exon 6		5′-TGTTTGTTTTGTGTGTGGGAAT-3′	5′-GCCAGGTAAACTAACAAGTAAAACC-3′
Exon 7		5′-TGGAAATAAGTTTGCTTTGAGAA-3′	5′-TGTTTTCTGAACGTCTGACTAGC-3′
Exon 17		5′-TGTCAGTGTTCTAAATATTTGTTTTGT-3′	5′-GGTGTACTGGTTAAAATGTTGGTTC-3′
Exon 18		5′-CAGATGCAGTTAAAATCTGTCAA-3′	5′-AAACTTTTAAAGGTCCAGCTTTTG-3′
Genomic DNA	SWSS2137	5′-GACAGGCTCAGATATTTC-3′	5′-CAACCATCTGTTGTCTTC-3′
mRNA		5′-AGCAACTATCACCAGAAGCACA-3′	5′-TTTTCCTGAATTTGAATCATGC-3′

*PHF14*, plant homeodomain finger protein 14; STS, sequence-tagged site.

**Table II:** Chromosomal regions that were amplified or homozygously deleted in BTC cell lines.

DNA copy number	Chromosomal region	Cell line	Known oncogene or tumor suppressor gene	Number of genes
Amplification	12p11.1-q11	NOZ		1
	12p12.1	NOZ	*KRAS*	8
	12q12	TKKK		1
	17q12	TKKK	*ERBB2*	35
	22q11.2	TKKK		20
Homozygous deletion	3p14.2^a^	TFK1, HuCCT1, OCUGI	*FHIT*	1
	5q12	OCUG1		1
	6q16.3-q21	OZ		2
	7p21.3	OZ		5
	9p21^a^	TFK1, OZ	*CDKN2A*, *CDKN2B*	3
	16q23.1	NOZ	*WWOX*	1
	20p12.1^a^	TFK1, OCUGI		1
	21q21.3	TKKK		1

a Common homozygous deletions. BTC, biliary tract cancer.
